# Editorial: Thymus research and development: a new look at the past, current knowledge, and future perspectives

**DOI:** 10.3389/fimmu.2026.1919476

**Published:** 2026-07-10

**Authors:** Valentin P. Shichkin, Miho Shinzawa, Dominik Filipp, Mariastefania Antica, Domenico V. Delfino

**Affiliations:** 1 Department of Biotechnology, Faculty of Health Sciences, National University “Kyiv Aviation Institute”, Kyiv, Ukraine; 2 OmniFarma LLC, Kyiv, Ukraine; 3 Experimental Immunology Branch, National Cancer Institute, National Institutes of Health, Bethesda, MD, United States; 4 Laboratory of Immunobiology, Institute of Molecular Genetics of the Czech Academy of Sciences, Prague, Czechia; 5 School of Dental Medicine, University of Zagreb, Zagreb, Croatia; 6 Foligno Nursing School, Department of Medicine and Surgery, University of Perugia, Perugia, Italy; 7 Section of Pharmacology, Department of Medicine and Surgery, University of Perugia, Perugia, Italy

**Keywords:** B cells, immunosenescence, radioresistant stem cells, T cells, thymus, thymus regeneration, thymus-associated pathologies

## 1 Introduction

Thymic research represents one of the most fascinating chapters in the history of immunology. Following the landmark work of Jacques Miller published in 1961, which established the thymus as the central organ of T-cell development and adaptive immunity (1, 2), the field evolved through periods of both enthusiasm and skepticism. For decades, the physiological importance of the thymus was in acute debates, partly due to the widespread practice of thymectomy during pediatric cardiac surgery (3, 4) hence casting doubt on the physiological importance of this organ (3–5). However, as early as 2010, new experimental techniques and corresponding clinical studies have led to the current view that the thymus is crucial in the production of a diverse and self-tolerant T-cell repertoire, the maintenance of immune homeostasis, and immune competence over the course of the human lifespan (6–8).

In recent years, thymus research has entered a new phase spearheaded by technological advances, including single-cell multi-omics, spatial transcriptomics, gene-editing approaches, stem-cell and organoid technologies, microphysiological systems, high-resolution imaging, artificial intelligence-assisted data analysis, and systems immunology (9, 10). These methodologies have enabled the unprecedented characterization of thymic cellular heterogeneity, developmental trajectories, stromal-hematopoietic interactions, and mechanisms underlying thymic regeneration, aging as well as dysfunction. At the same time, increasing recognition of the impact of thymic involution on immunosenescence, susceptibility to infection, vaccine responsiveness, autoimmunity, and cancer has renewed interest in therapeutic strategies aimed at preserving or restoring thymic function (11–14).

**Figure 1:**
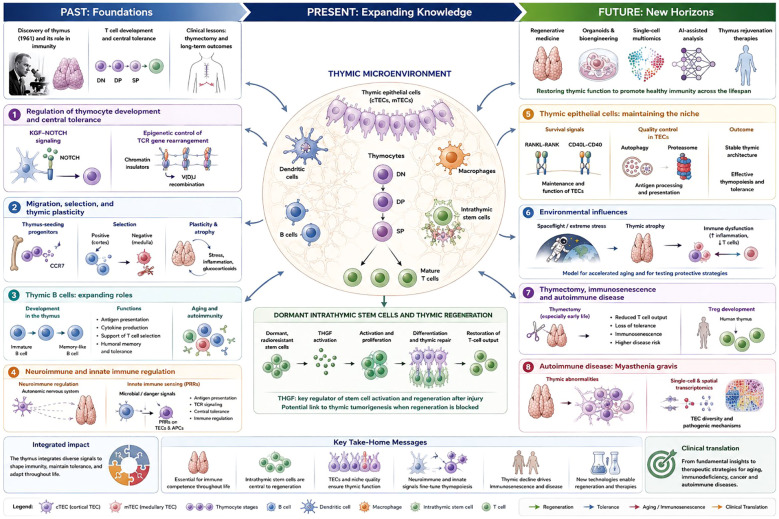
Thymus research and development: past, present, and future. APCs, antigen-presenting cells; DN, double negative; DP, double positive; KGF, keratinocyte growth factor; PRRs, pattern- recognition receptors; SP, single positive; TCR, T-cell receptor; TECs, thymic epithelial cells. Generative AI ChatGPT-5.2 (OpenAI) was used in the creation of this illustration in accordance with VS’s conceptual design and instructions.

This Research Topic is a compilation of original research articles and reviews that address the molecular, cellular, and systemic aspects of thymic biology that highlight the most recent and novel changes in the field. The contributions presented herein provide updated perspectives on thymic development, epithelial cell biology, regeneration, immune tolerance, aging, and thymus-associated disease, while highlighting emerging concepts and future directions for both basic and translational thymus research (**Figure 1**).

## 2 Regulation of thymocyte development and central tolerance

The primary function of the thymus is the generation of a diverse yet self-tolerant T-cell repertoire. Several contributions in this Research Topic explore the molecular mechanisms that are responsible for controlling thymocyte differentiation and selection.

Teng et al. showed that keratinocyte growth factor (KGF) regulates thymocyte development through modulation of Notch signaling. Their findings demonstrate how microenvironmental signals regulate developmental checkpoints and influence T-cell maturation.

In addition to the role of KGF in T cell development, Zhu et al. investigated the regulation of T-cell receptor diversity through chromatin organization and Tcrd gene recombination. Their work identified chromatin insulators, such as CTFC-cohesion complexes, as important regulators of V(D)J recombination, emphasizing the critical role of epigenetic mechanisms in shaping the adaptive immune repertoire.

Raposo et al. explored thymically committed regulatory CD4 T cells (tTregs), which were shown to be essential for immune homeostasis and self-tolerance. In this study, the authors described the human tTreg expression signature by comparing genome-wide transcriptomic profiles between tTregs and their conventional counterparts (tTconvs) and identified a subset of 250 genes significantly expressed only in the experimental tTregs. Their results provided a unique resource that will inform future studies of putative biomarkers for thymically committed Tregs.

Advances in understanding immune tolerance have been further illustrated by the work of Moleirinho et al., who employed sophisticated flow cytometric approaches that characterized regulatory T-cell development within the human thymus. Their findings provide valuable markers for tracking Treg ontogeny and may support future therapeutic strategies aimed at restoring tolerance following thymic injury or immune deficiency.

Together, these studies underscore the highly coordinated molecular networks that govern T-cell development and central tolerance.

## 3 Migration, selection, and thymic plasticity

The progression from hematopoietic progenitors to mature T cells involves tightly regulated migration, differentiation, and selection processes. The review by Perez et al. specified the pathways that guide thymus-seeding progenitors into the thymic microenvironment and direct their commitment toward the T-cell lineage.

The authors emphasized the importance of NOTCH signaling, extracellular matrix interactions, and thymic epithelial cell-mediated selection in determining T-cell fate. They also discussed the high plasticity of the thymus during periods of stress, inflammation, and endocrine imbalance.

Also in the Perez review, the discussion of the ability of the thymus to undergo acute atrophy in response to systemic inflammation while retaining the ability to regenerate and restore T-cell production after recovery was particularly noteworthy. These properties highlight the dynamic nature of the organ and its ability to adapt to changing physiological conditions.

## 4 Thymic epithelial cells and maintenance of the thymic niche

Thymic epithelial cells (TECs) constitute the structural and functional backbone of the thymic microenvironment. Therefore, maintenance of TEC integrity is therefore essential for effective thymopoiesis.

In a study conducted by Hayama et al., the complementary roles for RANK and CD40 signaling pathways in the preservation of TEC populations and support of their functional capacity in the adult thymus were identified. Their findings suggest potential targets for regenerative interventions aimed at restoring thymic architecture. It is also key that the authors emphasized the use of single cell RNA sequencing as part of modern advanced technologies utilized by researchers in the field.

Highlighting a focus on intracellular quality-control mechanisms, a review by Yamaguchi et al. examined the importance of autophagy and proteasome function in TEC biology. Beyond maintaining cellular homeostasis, these pathways have been shown to influence antigen processing and presentation, thereby contributing directly to thymocyte selection and immune tolerance. From their analysis of related studies, the authors proposed the distinctive perspective of autophagy in self-peptide generation and the autophagy activity regulation in TECs.

Together, these two studies placed emphasis the concept that successful regeneration of the thymus requires the preservation of both cellular composition and functional integrity of the thymic niche.

## 5 Radioresistant intrathymic stem cells, thymic regeneration and cancerogenesis

One of the central themes addressed in this Research Topic concerns the existence and biological significance of radioresistant intrathymic stem cells. Shichkin analyzed experimental evidence that support the presence of dormant stem-cell populations within the thymus that become activated by acute stressors such as irradiation that lead to thymic injury.

As a result of the analyzed experimental evidence, the author proposed a model that highlighted the potential role of autocrine thymocyte growth factor (THGF) as a regulator of these dormant cells, promoting their activation, proliferation, and differentiation during thymic regeneration. This analysis also revealed parallels between THGF-responsive cells and other radioresistant thymic populations, suggesting the existence of an integrated regenerative network involving both stromal and lymphoid compartments.

Beyond regeneration, this concept raises important questions regarding the involvement of dormant stem cells and disrupted regenerative pathways in thymic tumorigenesis. The hypothesis presented by Shichkin provide a framework for future experimental studies aimed at understanding the mechanisms of thymic repair and the nature of dormant cancer stem cells in the context of existence the natural radioresistant dormant stem cells.

## 6 Expanding roles of thymic B cells

Although traditionally viewed as a T-cell organ, the thymus contains functionally important B-cell populations that are increasingly recognized as contributors to immune regulation. The review by Wedemeyer and Griffith described the emerging role of thymic B cells in aging and autoimmunity, suggesting that age-related alterations in these populations may contribute to chronic inflammation and immune dysregulation.

Similarly, Castañeda et al. demonstrated that thymic B cells undergo early-life differentiation into memory-like subsets, indicating that the thymic microenvironment may contribute to humoral immune memory as well as tolerance induction.

These findings broaden the functional landscape of the thymus and challenge the traditional view of the organ as solely a site of T-cell development.

## 7 Neuroimmune and innate immune regulation of the thymus

In recent years, evidence has been found that shows that the thymus intricately operates with other organs as well, specifically cooperating with the complex network of neural and immune interactions.

Carpenter et al. have reviewed evidence demonstrating that autonomic nervous system inputs influence thymic epithelial cell maintenance, thymic involution, and regenerative responses.

This growing understanding of neuroimmune communication suggests potential therapeutic opportunities to delay thymic aging and improve recovery following injury.

Mukherjee et al. explored the role of pattern-recognition receptors (PRRs) in thymic function. Their review highlights how microbial and endogenous danger signals influence antigen presentation, T-cell receptor signaling, and central tolerance mechanisms.

These studies illustrate how the thymus integrates information from both the nervous and innate immune systems to regulate adaptive immunity.

## 8 Environmental influences on thymic function

External environmental factors can profoundly affect thymic structure and function. Muramatsu et al. used spaceflight-induced thymic atrophy as a model of accelerated thymic aging and immune dysfunction.

This review provides unique insights into mechanisms of thymic involution under extreme physiological stress and may contribute to the development of interventions designed to preserve thymic function during aging, chronic disease, or prolonged environmental challenges.

## 9 Thymectomy, immunosenescence, and autoimmune diseases

The long-term consequences of thymectomy remain a topic of considerable clinical importance. Tsirkin et al. provide evidence linking thymectomy with increased susceptibility to immune dysregulation, chronic disease, and accelerated immunosenescence.

Their findings support growing concerns that the removal of the thymus, particularly early in life, may have lifelong consequences for immune competence and disease susceptibility. These observations underscore the need for careful evaluation of thymectomy practices and consideration of long-term immunological outcomes.

In regard to modern approaches currently utilized in immunology, Matsui et al. review on myasthenia gravis, which is an autoimmune disease strongly associated with thymic abnormalities summarized recent advances in single-cell and spatial transcriptomic analyses that have revealed previously unrecognized diversity among thymic epithelial cell populations.

These findings provide new perspectives on the cellular mechanisms underlying thymic pathology in myasthenia gravis and identify potential directions for biomarker development and therapeutic intervention.

## 10 Conclusion

The compilation of studies assembled in this Research Topic demonstrate that the thymus is a dynamic and multifunctional organ that remains essential for the maintenance of immune competence throughout life.

Several overarching themes unite these contributions: the importance of intrathymic stem cells in regeneration, the precise molecular regulation of thymocyte development and tolerance, the expanding functional significance of thymic B cells, the influence of neuroimmune and innate immune signaling, and the critical role of thymic epithelial cells in maintaining a supportive microenvironment.

Importantly, the recent evidence highlights the clinical consequences of thymic dysfunction, involution, and removal. At the same time, advances in stem-cell biology, regenerative medicine, systems immunology, and high-resolution cellular analysis are creating promising opportunities to restore thymic function and improve immune health.

By bridging fundamental biology and clinical medicine, research on the thymus remains central to our understanding of immunity, aging, autoimmunity, and the development of regenerative therapies for the immune system.
